# TMEM30A is a candidate interacting partner for the β-carboxyl-terminal fragment of amyloid-β precursor protein in endosomes

**DOI:** 10.1371/journal.pone.0200988

**Published:** 2018-08-07

**Authors:** Nobumasa Takasugi, Runa Araya, Yuji Kamikubo, Nanaka Kaneshiro, Ryosuke Imaoka, Hao Jin, Taku Kashiyama, Yoshie Hashimoto, Masaru Kurosawa, Takashi Uehara, Nobuyuki Nukina, Takashi Sakurai

**Affiliations:** 1 Department of Cellular and Molecular Pharmacology, Juntendo University Graduate School of Medicine, Bunkyo-ku, Tokyo, Japan; 2 Department of Medicinal Pharmacology, Graduate School of Medicine, Dentistry, and Pharmaceutical Sciences, Okayama University, Okayama, Japan; 3 Laboratory for Structural Neuropathology, RIKEN Brain Science Institute, Saitama, Japan; 4 Laboratory of Structural Neuropathology, Doshisha University Graduate School of Brain Science, Kyoto, Japan; 5 Department of Neuroscience for Neurodegenerative Disorders, Juntendo University Graduate School of Medicine, Bunkyo-ku, Tokyo, Japan; Torrey Pines Institute for Molecular Studies, UNITED STATES

## Abstract

Although the aggregation of amyloid-β peptide (Aβ) clearly plays a central role in the pathogenesis of Alzheimer’s disease (AD), endosomal traffic dysfunction is considered to precede Aβ aggregation and trigger AD pathogenesis. A body of evidence suggests that the β-carboxyl-terminal fragment (βCTF) of amyloid-β precursor protein (APP), which is the direct precursor of Aβ, accumulates in endosomes and causes vesicular traffic impairment. However, the mechanism underlying this impairment remains unclear. Here we identified TMEM30A as a candidate partner for βCTF. TMEM30A is a subcomponent of lipid flippase that translocates phospholipids from the outer to the inner leaflet of the lipid bilayer. TMEM30A physically interacts with βCTF in endosomes and may impair vesicular traffic, leading to abnormally enlarged endosomes. APP traffic is also concomitantly impaired, resulting in the accumulation of APP-CTFs, including βCTF. In addition, we found that expressed BACE1 accumulated in enlarged endosomes and increased Aβ production. Our data suggested that TMEM30A is involved in βCTF-dependent endosome abnormalities that are related to Aβ overproduction.

## Introduction

Alzheimer’s disease (AD) is a progressive neurodegenerative disorder that is characterized by senile plaques and neurofibrillary tangles. Amyloid-β (Aβ), the major component of senile plaques, is produced from the sequential cleavage of Aβ precursor protein (APP) by β-site APP-cleaving enzyme 1 (BACE1) and γ-secretase [1]. Numerous genetic and biochemical studies support the hypothesis that AD pathology is associated with Aβ overproduction and aggregation (amyloid hypothesis) [2]. However, increasing evidence suggests that Aβ-independent abnormalities precede Aβ depositions [3].

Abnormally enlarged neuronal endosomes, a sign of retrograde traffic impairment, have been observed in the early phase of AD and in Down syndrome (DS), which exhibits progressive AD-like dementia [4, 5]. The retrograde traffic pathway recycles endocytosed proteins from endosomes to the trans-Golgi network (TGN) or plasma membrane, and the impairment of this pathway results in APP and BACE1 accumulation in endosomes to overproduce Aβ [6, 7]. In DS, trisomy of chromosome 21, which contains the APP gene, increases APP metabolites and induces endosomal traffic impairments [4]. Further analysis using fibroblasts derived from patients with DS demonstrated that endosome enlargement depends on the β-cleaved C-terminal fragment of APP (βCTF), which is a BACE1 cleavage product, and not on the Aβ [8]. In the brains of patients with AD, besides the upregulation of BACE1 activity during disease progression [9, 10], βCTF accumulates [11]. However, the mechanisms underlying βCTF accumulation and its effect on morphological changes in endosomes remain largely unknown.

TMEM30 (CDC50) family proteins are conserved across species and interact with type 4 P-type ATPase (P4-ATPase) to form lipid flippases [12]. Lipid flippases translocate phospholipids such as phosphatidylserine (PS) and phosphatidylethanolamine from the outer to the inner leaflet of the lipid bilayer. TMEM30 family proteins are required for P4-ATPase, which is the catalytic component for lipid flippase, to exit the endoplasmic reticulum (ER) and become stabilized [13–15]. Proper PS localization in endosomes is required for the endosomal traffic to assemble PS-binding proteins such as EHD1 and evectin-2 [16, 17] and to form the curvature of sorting vesicles for budding [18]. Studies with *Caenorhabditis elegans* revealed that the ablation of TMEM30 and P4-ATPase orthologs (CHAT-1 and TAT-1, respectively) results in PS mislocalization and impairment of endocytic sorting and recycling [19, 20]. A recent study suggested TMEM30A and TMEM30B to be candidate Aβ receptors on the cell surface [21]. However, the involvement of TMEM30 in endosomal abnormalities remains unclear.

In this study, we report that TMEM30A, the major isoform of TMEM30 family proteins in neuronal cells, is a likely partner for βCTF in impairing vesicular traffic and forming abnormally enlarged endosomes. Thus, our results shed light on unidentified molecular mechanisms of βCTF-mediated AD pathogenesis.

## Materials and methods

### Production of polyclonal antibodies against TMEM30A

For polyclonal antibody production against TMEM30A and ATP8A1, peptides with amino acid sequence CKYRNSSNTADITI (the C-terminal 13 residues of mouse TMEM30A plus an N-terminal Cys) and CRAYDTTKQRPDEW (the C-terminal 13 residues of human ATP8A1 plus an N-terminal Cys) were synthesized, conjugated with keyhole limpet hemocyanin and injected into Japanese White rabbits (Kitayama Labes). The antisera were affinity purified with the antigen polypeptide immobilized on a coupling gel (SulfoLink; Thermo Fisher Scientific). All experiments were carried out in accordance with the institutional guidelines on animal experimentation and were approved by the Juntendo University Animal Care and Use Committee (approval number: 200069). All efforts were made to minimize suffering and distress of the animals. Intracardiac puncture for the whole blood collection was performed under deep terminal anesthesia with sodium pentobarbital.

### Compounds, antibodies, and immunological methods

The γ-secretase inhibitor N-[N-(3,5-difluorophenacetyl-L-alanyl)]-(S)-phenylglycine t-butyl ester (DAPT) (565770, Merck Millipore) was solubilized in DMSO. The synthetic Aβ40 peptide (4307-v), and LysoTracker Red DND-99 (L7528) were purchased from Peptide Insititute Inc and Thermo Fisher Scientific, respectively. The following antibodies were used: rabbit anti-APP C-terminal (APPC15) [22], mouse anti-APP N-terminal (clone 22C11), mouse anti-APP C-terminal [clone mC99 (70–80)] (Millipore), mouse anti-human Aβ/βCTF (clone 82E1, N-terminal-end specific), rabbit anti-BACE1 (C), rabbit anti-APP (C), rabbit anti-sAPPβ-wild type (Immuno-Biological Laboratories), mouse anti-human Aβ (clone 6E10; Covance), mouse anti-α-Tubulin (clone DM1A; Sigma-Aldrich), mouse anti-Rab-5 (clone D-11), mouse anti-Myc-tag (clone 9E10) (Santa Cruz Biotechnology), mouse anti-green fluorescent protein (GFP) (for immunoprecipitation, clone 3E6; Life Technologies), mouse anti-GFP (clone GF200; Nacalai Tesque), rabbit anti-Rab7 (clone D95F2), rabbit anti-Rab11 (clone D4F5) (Cell Signaling Technology), mouse anti-GM130 (clone 35/GM130; BD Bioscience). For secondary antibodies, HRP-linked sheep anti-mouse IgG (NA931) and donkey anti-rabbit IgG (NA934) (GE Healthcare) were used for immunoblotting. Goat anti-mouse IgG (H+L) conjugated with Alexa Fluor 488 (A-11029) or Alexa Fluor 594 (A-11032) and goat anti-rabbit IgG (H+L) conjugated with Alexa Fluor 488 (A-11034) or Alexa Fluor 594 (A-11037) (highly-cross adsorbed; Thermo Fisher Scientific) were used for immunocytochemical analysis. To detect Aβ, medium samples were analyzed by performing human Aβ-specific ELISA (#27729, Immuno-Biological Laboratories) or immunoblotting.

### DNA constructs

The mammalian expression constructs of human APP695 wild type-myc and APP Swedish mutation-myc, and human BACE1 were previously described [22]. pcDNA3-SC100 [23] was a kind gift from Dr. Kei Maruyama (Saitama University Medical School). Rat TGN38 cDNA was subcloned into pDsRed-N1. The coding sequences for mouse TMEM30A, KKLN mutant, and ATP8A1 were cloned into Gateway entry vectors (pENTR/D-TOPO or pENTR-1A, Thermo Fisher Scientific), and subcloned into pcDNA-DEST40, pDEST-CFP, or pDEST-mCherry vectors to express untagged proteins or with their N-terminus fused to CFP or mCherry using Gateway LR Clonase (Thermo Fisher Scientific). The expression vectors for truncated mutants of human APP were prepared from pcDNA3-APP-Venus. The coding sequence of the extracellular region of TMEM30A (TmEx; 67–323 AA) was amplified using PCR and was cloned into a pENTR/D-TOPO vector. The target sequence was subcloned into pDEST-15 to develop a GST fusion protein. pGEX-6P-1 (GE Healthcare) was used for the control experiments. Each insertion was confirmed using DNA sequencing.

### Cell culture and transfection

The African green monkey kidney fibroblast cell line COS-7 was obtained from RIKEN BRC Cell Bank (Tsukuba) and cultured in Dulbecco's modified Eagle's medium supplemented with 10% fetal bovine serum, 100 units/ml penicillin, 100 μg/ml streptomycin, and 2 mM glutamine (Nacalai Tesque). For plasmid transfection, Lipofectamine^™^ 2000 (Life Technologies) was used for biochemical analysis and Fugene HD (Promega) was used for immunocytochemical analysis as previously described [24–27].

### Immunoblotting

Cells were lysed with RIPA buffer (50 mM Tris-HCl (pH8.0), 150 mM sodium chloride, 0.5% sodium deoxycholate, 0.1% sodium dodecyl sulfate, 1% NP-40) containing cOmplete protease inhibitor cocktail (Roche). For standard immunoblotting analysis, aliquots of lysates were separated on 12% Tris-glycine gels as previously described [25, 26]. For the detection of sAPPα and sAPPβ, aliquots of conditioned media were separated by 10% Tris-glycine gels, and probed with 6E10 and anti-sAPPβwt antibody, respectively. For the detection of APP-CTFs and Aβ, high-resolution electrophoresis was performed using 16% Tris-Tricine gels. APP-CTFs were detected with APPC15. The β1CTF and Aβ were detected by 82E1. The separated proteins on a membrane, were incubated with appropriate primary and secondary antibodies, and detected with ImmunoStar LD (Wako Pure Chemical Industries). For quantification chemiluminescence light signals were captured by a cooled charge-coupled device camera system (LAS-3000plus; Fuji Film).

### Immunocytochemical analysis

Cells cultured on glass coverslips were fixed for 30 min in 4% paraformaldehyde, and then permeabilized and blocked for 30 min with 0.1% Triton X-100 and 3% bovine serum albumin. All solutions were made in phosphate buffered saline (PBS). Coverslips were then incubated with primary antibodies as indicated for overnight at 4°C. After washing with PBS, coverslips were incubated with secondary antibodies for 1 h at room temperature, washed with PBS, and mounted on slide glass using PermaFluor Aqueous Mounting Medium (Thermo Fisher Scientific). Fluorescent images were captured by the following inverted microscopes, IX71 (Olympus), BZ-8100 (Keyence), and FSX100 (Olympus), using manufacturer’s software. For counting of intracellular APP-containing vesicles, images of randomly selected microscopic fields were captured using a 100x objective lens. The APP-containing vesicles in eight fields were counted in each experiment, and the experiments were performed in triplicate. Each image was automatically thresholded and processed by ImageJ particle analysis plugin.

### Immunoprecipitation and GST pulldown assay

Cells were solubilized with 1% CHAPS lysis buffer [50 mM HEPES, pH 7.4, 150 mM NaCl, 1 mM EDTA supplied with protease inhibitor cocktail (Roche)]. Lysates were clarified by centrifugation at 12,000 ×g for 5 min, and the supernatants were incubated with antibodies with gentle rocking at 4°C overnight. Protein A or G agarose beads (GE Healthcare) were pre-equilibrated with ice-cold lysis buffer, applied to the protein–antibody mixture, and incubated for another 1–2 h at 4°C. For immunoprecipitation, mouse IgG2a (M9144, Sigma-Aldrich) or IgG1 (M3198, Sigma-Aldrich) was used as a control. The beads were then centrifuged down and washed thrice with the lysis buffer. For GST pulldown assays, cells were lysed in 1% CHAPS lysis buffer on ice for 30 min. Lysates or synthetic Aβ40 diluted with lysis buffer were clarified by centrifugation for 5 min at 12,000 × g, and incubated with GST-fusion proteins pre-bound to GSH-agarose beads (GE Healthcare) for 2 h at 4°C. The precipitates were solubilized in Laemmli sample buffer and subjected to immunoblotting analysis.

### Statistical analysis

Data are presented as mean, and error bars indicate SEM. The treatment groups were compared using one-way ANOVA with Tukey’s post hoc test. Significance was set at *P < 0.05, **P < 0.01; and ***P < 0.001. Differences >0.05 were considered not significant (N.S.).

## Results

### Induction of endosomal enlargement and accumulation of βCTF by the coexpression of TMEM30A and APP

**Fig 1:**
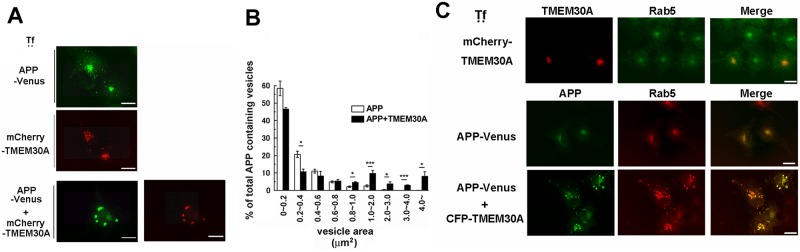
Coexpression of TMEM30A and APP induces enlarged endosomes. A, B: COS-7 cells were transfected with APP-Venus and mCherry-TMEM30A. (A) Coexpression of APP and TMEM30A resulted in the redistribution of these proteins in enlarged vesicles. Scale bar: 20 μm. (B) The distribution of APP-containing vesicles in transfected cells classified by their size. The average number was evaluated by eight fields (×100, 8–11 cells), with counting in each experiment [independent experiment performed thrice (*n* = 3), mean ± SEM, *P < 0.05, ***P < 0.001]. Auto-thresholded images were processed using Image J. (C) Upper panel: COS-7 cells were transfected with mCherry-TMEM30A. Cells were labeled with anti-Rab5 antibody (green). Lower panel: COS-7 cells were transfected with APP-Venus and CFP-TMEM30A. Cells were labeled with early endosome marker, anti-Rab5 (red). Scale bar: 20 μm.

To examine the effects of TMEM30A on APP distribution, we transfected COS-7 cells with APP-Venus and mCherry-TMEM30A. Single transfection of APP or TMEM30A mainly demonstrated perinuclear localization with punctate vesicular structures throughout the cells, which coincides with their reported distributions in TGN and endosomes [28, 29] (Fig 1A). The cotransfection of TMEM30A and APP dramatically altered the distribution of both proteins to aberrantly large vesicular structures (Fig 1A). Morphometric comparison revealed that cotransfection with TMEM30A enlarged APP-containing vesicles to >0.8 μm^2^, which was rarely observed in singly transfected cells (Fig 1B). Further, we showed that the cotransfection of APP and TMEM30A resulted in a significant codistribution of the early endosome marker Rab5 and APP in enlarged vesicles (Fig 1C, and Panel A in S1 Fig), suggesting the endosomal features of these vesicles. The colocalization of APP and Rab5 was confirmed by a different antibody (Panel B in S1 Fig). By using cotransfection, we also observed colocalization of TMEM30A and APP in similar large vesicular structures in rat primary hippocampal neurons (S2 Fig).

### Complex formation of TMEM30A and APP-βCTF in endosomes

**Fig 2:**
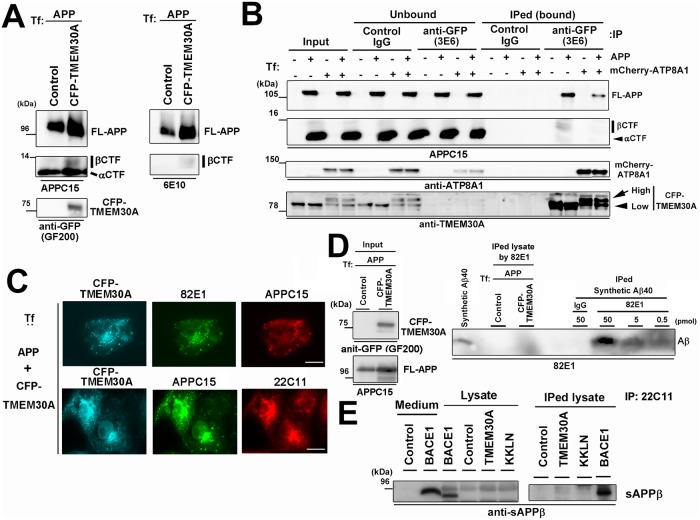
TMEM30A interacts with βCTF in endosomes. A: APP and CFP-TMEM30A were cotransfected into COS-7 cells. The effects of TMEM30A expressions on FL-APP and APP-CTFs were analyzed using immunoblotting of cell lysates. 6E10, a monoclonal antibody against N-terminal portion of Aß, was used for ßCTF detection. B: APP, CFP-TMEM30A, and mCherry-ATP8A1 were transfected into COS-7 cells. Coimmunoprecipitation analysis of COS-7 cell lysates was performed using control mouse IgG or GFP antibody (3E6) to precipitate CFP-TMEM30A. C: COS-7 cells were transfected with APP and CFP-TMEM30A. Upper panel: Cells were labeled with Aβ/βCTF-specific antibody (82E1, green) and APP C15 (red). Lower panel: Cells were labeled with the APP C15 (green) and 22C11, (red). Scale bar: 20 μm. D: Analysis of intracellular Aβ accumulation of APP and TMEM30A-transfected COS-7 cells. 48 h after transfection, cells were lysed and the lysate containing 2 mg protein was immunoprecipitated with 82E1. Control was performed by adding indicated amounts of synthetic Aβ40 to the lysis buffer followed by immunoprecipitation. E: Analysis of intracellular sAPPβ accumulation in COS-7 cells transfected with APP and TMEM30A or BACE1. 48 h after transfection, cells were lysed and the lysate containing 2 mg protein was immunoprecipitated with 22C11. Precipitates were analyzed using the sAPPβ-specific antibody.

APP is cleaved by β- and γ-secretases to produce the secreted form of APPβ (sAPPβ), βCTF, and Aβ. Alternatively, APP is cleaved by α- and γ-secretases to produce sAPPα, αCTF, and P3 [30]. APP metabolites produced by cleavages of secretases and antibodies used to detect each metabolite are summarized in S3 Fig. The cotransfection of APP and TMEM30A increased full length APP (FL-APP), αCTF, and βCTF (Fig 2A). The variability in level of expression among each protein in cotransfection experiments might affect our results. For this reason, we performed simple transfection with CFP-TMEM30A in human neuroblastoma BE(2)-C cells and analyzed endogenous APP. Intriguingly, the expression of CFP-TMEM30A caused an accumulation of APP-CTFs derived from endogenous APP (Panel A in S4 Fig). To analyze the interaction between APP and TMEM30A, immunoprecipitation was performed using human neuroblastoma SH-SY5Y cells, which stably express Swedish mutant of APP to increase ßCTF. Endogenous TMEM30A was coprecipitated with APP-CTF (Panel B in S4 Fig). These data may suggest that APP-CTF and TMEM30A form a complex in conditions relevant to neurons. Next, we analyzed the influence of APP on formation of the lipid flippase complex. We transfected COS-7 cells with CFP-TMEM30A, APP, and mCherry-fused ATP8A1 (mCherry-ATP8A1), the brain-enriched lipid flippase subunit that interacts with TMEM30A [28], and coimmunoprecipitated TMEM30A-interacting proteins. Although αCTF was the most abundant APP-CTFs in the input sample, TMEM30A seemed to interact mainly with FL-APP and βCTF but less with αCTF (Fig 2B). ATP8A1 expressions facilitated TMEM30A glycosylation as previously reported [31], suggesting the formation of a lipid flippase complex. ATP8A1 expression concomitantly reduced the interactions between TMEM30A and FL-APP and between TMEM30A and βCTF (Fig 2B). Although interactions with other P4-ATPases remain possible, our results suggest that TMEM30A and APP metabolites form a complex that is independent of lipid flippase. Next, using specific antibodies, we attempted to identify APP metabolites associated with TMEM30A in enlarged endosomes. The enlarged endosomes were recognized by antibody 82E1, which reacts with the cleaved end of β-secretase and specifically detects the Aβ or βCTF [32] and antibody APPC15, which recognizes FL-APP and APP-CTFs. However, the APP N-terminal antibody (22C11), which detects FL-APP and sAPP, recognized enlarged vesicles with a weak manner (Fig 2C). These results suggested that βCTF may be a major fragment of APP in enlarged endosomes. To further explore the involvement of other APP cleavage products, we tried to detect intracellular accumulation of Aβ and sAPPβ in TMEM30A and APP-cotransfected cells. Although 82E1 enabled the immunoprecipitation of synthetic Aβ40, it failed to detect Aβ in the cell lysate (Fig 2D). Similarly, the 22C11 immunoprecipitated sAPPβ from the lysate of cells transfected with BACE1 and APP but not from those transfected with TMEM30A and APP (Fig 2E). Although APP metabolites, even below their detection limit, may be responsible for impaired vesicular traffic, our data suggested that the interaction between TMEM30A and βCTF in endosomes might be responsible for the endosomal enlargement.

### TMEM30A interacts with the N-terminus and Aβ regions of APP

**Fig 3:**
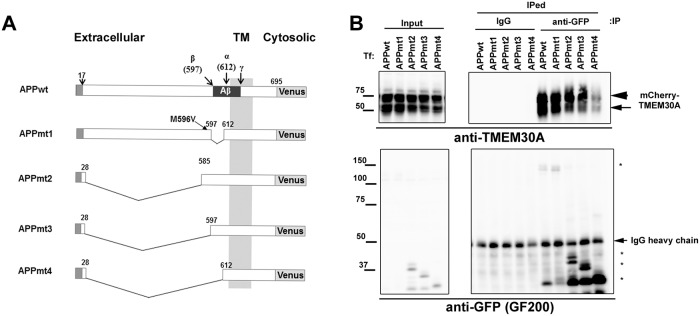
TMEM30A interacted with APP at the APP N-terminal domain and Aβ N-terminal sequence. A: Schematic depiction of deletion mutants of APP-Venus used in this study. TM; Transmembrane domain. B: COS-7 cells were transfected with mCherry-TMEM30A and the wild-type or deletion mutant of APP-Venus. After 24 h, the medium was replaced with a fresh medium containing a γ-secretase inhibitor (10 μM DAPT) and further incubated for 24 h. (B) Coimmunoprecipitation analysis of COS-7 cell lysates using control mouse IgG or GFP antibody (3E6) to precipitate APP-Venus. The arrowhead, arrow, and asterisks represent mature, immature mCherry-TMEM30A, and deletion mutants of APP-Venus, respectively.

To identify the interaction domain of APP with TMEM30A, we constructed a series of mutants of APP-Venus (Fig 3A), which have a mutated β-secretase cleavage site and deleted Aβ-N-terminal sequence [APPmt1 (M596V/Δ597–612)], a deletion of the extracellular region before the Aβ sequence [APPmt2 (Δ28–585), APPmt3 (Δ28–597)], and a deletion of the extracellular region, including the Aβ N-terminal sequence [APPmt4 (Δ28–612)]. We transfected COS-7 cells with mCherry-TMEM30A and mutants of APP-Venus, treated them with a γ-secretase inhibitor, DAPT, to accumulate CTFs, and performed immunoprecipitation using the anti-GFP antibody. We found that only APPmt4 (Δ28–612) decreased the coimmunoprecipitation of TMEM30A (Fig 3B). Considering that TMEM30A interacts with βCTF but less with αCTF (Fig 2B), TMEM30A may interact with APP in at least two regions, namely the N-terminal side from the β cleavage site and the Aβ N-terminal sequence.

### TMEM30A interacts with β1 and β11 CTFs, and their interaction is dependent on codistribution

**Fig 4:**
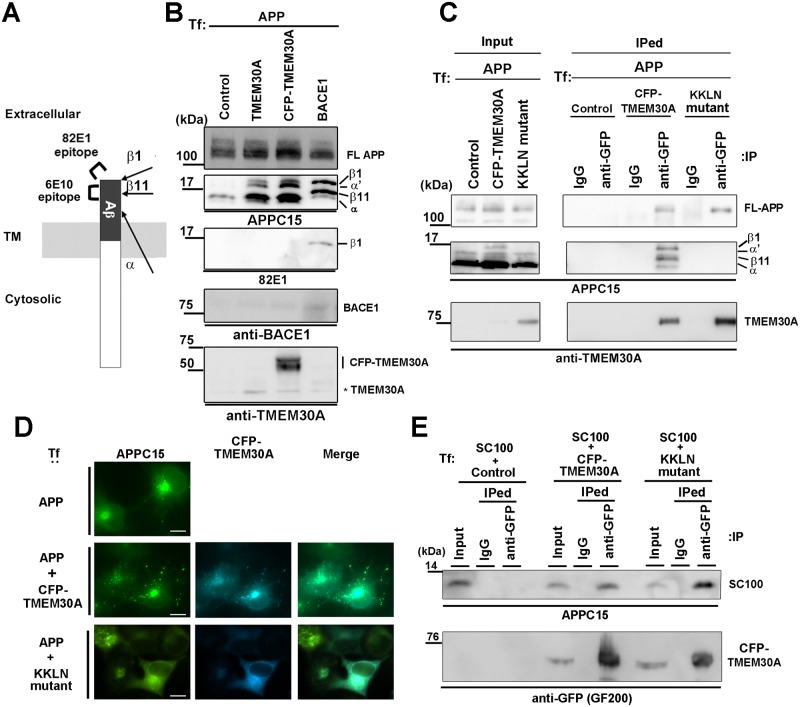
Intracellular interaction between βCTF and TMEM30A. A: Schematic depiction of APP-CTFs and their specific antibodies. TM; Transmembrane domain. B: COS-7 cells were transfected with APP and CFP-TMEM30A, untagged TMEM30A, or BACE1. To segregate β1- and β11-CTFs, immunoblot analysis was performed using high-resolution electrophoresis. β1CTF was detected by 82E1, which is specific for β1 cleaved end. Because BACE1 activity competes with α-secretase [32], α′CTF appeared to be derived from α-secretase cleavage. C, D: COS-7 cells were transfected with APP and the wild-type or KKLN mutant of CFP-TMEM30A. (C) Coimmunoprecipitation analysis of COS-7 cell lysates using control mouse IgG or GFP antibody to precipitate CFP-TMEM30A. (D) Immunofluorescence analysis. Cells were labeled with APPC15 antibody (green). Scale bar: 20 μm. E: Coimmunoprecipitation analysis of COS-7 cells that were transfected with artificial βCTF, SC100, and wild-type or KKLN mutant of CFP-TMEM30A using control mouse IgG or GFP antibody.

To further characterize TMEM30A-APP interaction, we analyzed APP-CTFs associated with TMEM30A using high-resolution electrophoresis. APP is cleaved by β-secretase at two different sites to produce β1 and β11 CTFs [33] (Fig 4A). Cotransfection with APP and BACE1 increased β1-CTF and β11-CTF but decreased FL-APP, αCTF, and α′CTF (Fig 4B). The transfection of CFP-tagged or untagged TMEM30A showed an increasing trend of FL-APP and APP-CTFs (Fig 4B), as observed in Fig 2A. TMEM30A is localized in plasma membranes and endosomes [28], while βCTF is produced in endosomes [29], suggesting that their codistribution in endosomes is critical for their interaction. To analyze this possibility, we created a TMEM30A mutant with an ER retention signal KKLN at its C-terminus. We performed transfection with APP and wild-type or KKLN mutant of TMEM30A and analyzed their interactions (Fig 4C). Wild-type TMEM30A interacted with β1- and β11-CTFs and FL-APP but not with αCTFs. The KKLN mutant bound to FL-APP but did not interact with APP-CTFs. Our results strongly suggested that TMEM30A interacted with both βCTFs in the late secretory pathway and in endosomes. Next, we analyzed the effects of the KKLN mutant on APP distribution. Although wild-type TMEM30A accumulated APP in intracellular vesicles, the KKLN mutant was codistributed with APP in ER-like reticular structures but not in vesicular structures (Fig 4D). Thus, both wild-type and KKLN mutant of TMEM30A can interact with artificial βCTF, SC100, which is distributed in ER and TGN [23] (Fig 4E). Our data strongly suggested that TMEM30A interacted with βCTF and that colocalization in endosomes is critical for their effects.

**Fig 5:**
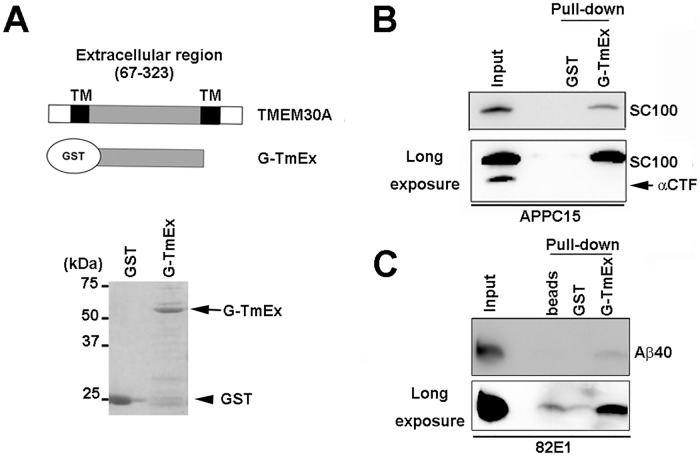
Direct interaction of βCTF with the extracellular region of TMEM30A. GST pulldown assay. (A) Upper panel: schematic view of recombinant TMEM30A extracellular region (67–323 AA) fused with GST (G-TmEx). Lower panel: Purified proteins were shown by Coomassie brilliant blue staining. (B) Lysate of COS-7 cells transfected with SC100 or (C) synthetic Aβ40 (1 μM) were incubated with recombinant GST or G-TmEx prebound to GSH-Sepharose.

To further analyze the interaction mode of APP and TMEM30A, we performed a GST pulldown assay using a GST protein that was fused with the extracellular region of TMEM30A (G-TmEx; Fig 5A). The extracellular region interacted with SC100 but not with its by-product αCTF (Fig 5B). Consistent with TMEM30A being a candidate for the Aβ receptor [21], G-TmEx pulled-down synthetic Aβ40 (Fig 5C), suggesting that the extracellular region of TMEM30A directly interacted with the Aβ N-terminal sequence.

### TMEM30A expression reduces β-secretase cleavage of APP but increases APP-CTFs

**Fig 6:**
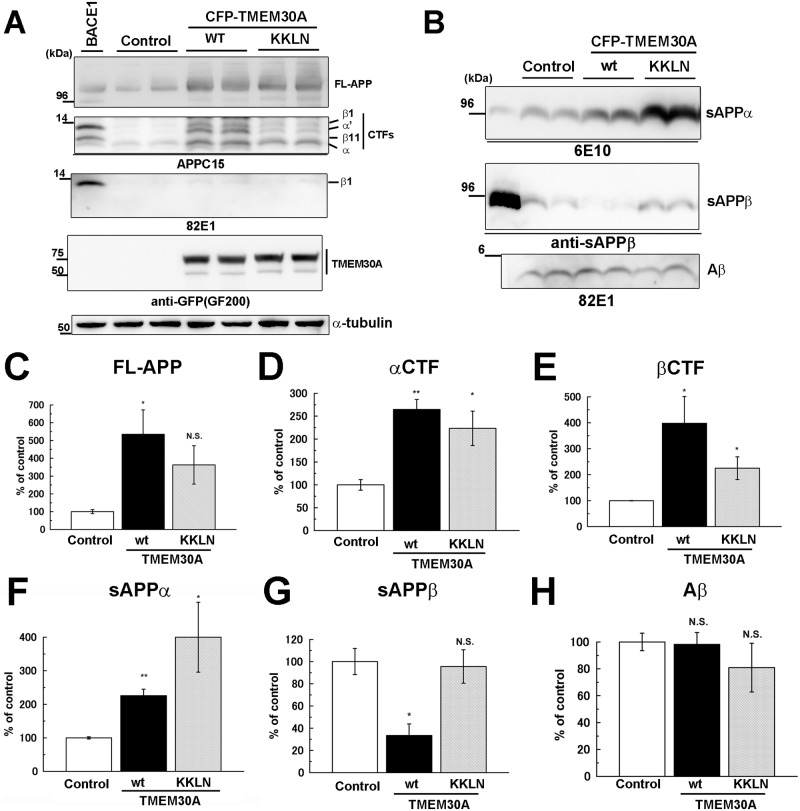
Coexpression of TMEM30A and APP decreased sAPPβ production but increased FL-APP and APP-CTFs. A, B: COS-7 cells were transfected with APP and the wild-type or KKLN mutant of TMEM30A or BACE1. After 24-h transfection, the medium was replaced, and the cells were further incubated for 24 h. Immunoblot analyses of cell lysate (A) and medium (B) are shown. C–H: Quantitative analyses are shown as follows and compared with control: (C) FL-APP, (D) αCTF, (E) βCTF, (F) sAPPα, (G) sAPPβ, and (H) Aβ [independent experiments were performed four times (*n* = 4), mean ± SEM, *P < 0.05, **P < 0.01; N.S., no significant difference].

We quantitatively analyzed the effect of exogenous TMEM30A on APP metabolism. The coexpression of TMEM30A and APP in COS-7 cells resulted in the accumulation of FL-APP (Fig 6A and 6C), αCTF (Fig 6A and 6D), and βCTF (Fig 6A and 6E). Compared with the wild-type, the KKLN mutant showed a decreasing trend of FL-APP and APP-CTF accumulation. The cotransfection of TMEM30A increased sAPPα (Fig 6B and 6F), but, seemingly inconsistent with the accumulation of APP-CTFs, it reduced sAPPβ (Fig 6B and 6G). In contrast, the KKLN mutant increased sAPPα but failed to alter sAPPβ (Fig 6B, 6F and 6G). The coexpression of the wild-type or KKLN mutant of TMEM30A failed to change Aβ production (Fig 6B and 6H). While sAPPβ produced in endosomes needs to be transported by a recycling pathway to the cell surface for secretion, sAPPα is generated mainly on the cell surface. One explanation for the decrease in sAPPβ secretion may be a defective secretion pathway. However, since we failed to detect intracellular sAPPβ accumulation (Fig 2E), an impairment of the secretion pathway was considered unlikely. These data may indicate that complex formation with TMEM30A reduces BACE1 cleavage of APP and that the accumulation of βCTFs occurs via independent mechanisms.

### Interaction between TMEM30A and APP/βCTF impairs endosomal traffic and maturation

**Fig 7:**
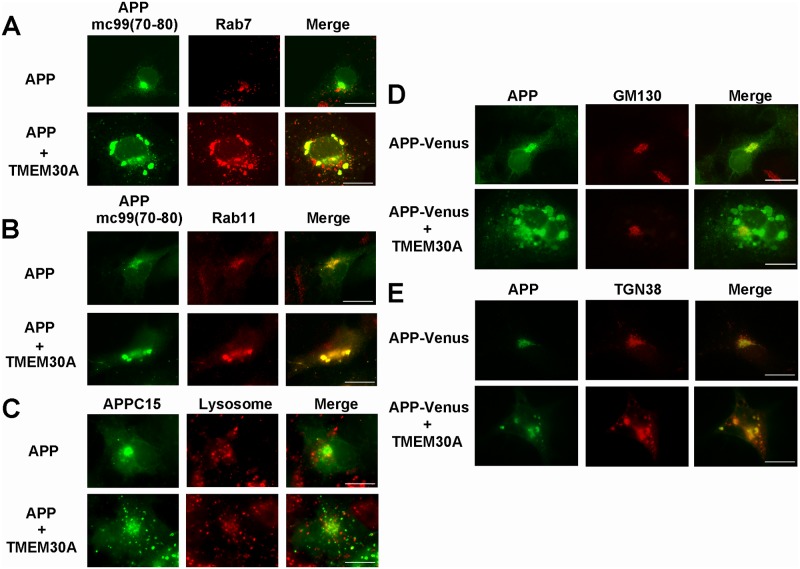
Expression of TMEM30A and APP impaired endosomal traffic and maturation. A–C: COS-7 cells were transfected with APP and CFP-TMEM30A, and immunofluorescently stained after 48 h of transfection. Cells were colabeled with anti-APP C-terminus [mC99 (70–80)] (green) and (A) anti-Rab7 or (B) anti-Rab11. In (C), cells were colabeled with APPC15 (green) and lysosome marker (LysoTracker) (red). D: COS-7 cells were transfected with CFP-TMEM30A and APP-Venus. Cells were labeled with anti-GM130 (red). E: COS-7 cells were transfected with CFP-TMEM30A and APP-Venus. After 24 h of transfection, DsRed-TGN38 was transfected, and the cells were further incubated for 24 h. Scale bar: 20 μm (A-E).

Using iodixanol discontinuous density gradient fractionation, we analyzed the effects of stable expression of Swedish mutant of APP on the distribution of endogenous TMEM30A (Panel C in S4 Fig). Interestingly, the distribution of TMEM30A was altered and showed a similar pattern to that of Rab5, and an overlap with that of APP-CTFs. These data are consistent with the interaction between APP-CTF and TMEM30A (Panel B in S4 Fig) in endosomes. We further characterized the enlarged endosomes produced by the coexpression of TMEM30A and APP by staining then with various organelle markers and small Rab GTPases such as Rab5 (early endosome), Rab7 (late endosome), and Rab11 (recycling endosome). Although Rab7 and Rab11 only partly colocalized with APP, the cotransfection of TMEM30A dramatically accumulated these Rab proteins in enlarged APP-containing vesicles (Fig 7A and 7B). Conversely, these enlarged vesicles rarely colocalized with a lysosome marker (Fig 7C). We also observed that mCherry tagged Rab5, Rab7, and Rab11 also accumulated in enlarged vesicles (S5 Fig). To further monitor retrograde traffic events, we expressed DsRed-tagged TGN38, which mainly localizes in TGN in the steady-state condition, but rapidly cycles between TGN and endosomes by retrograde traffic [34]. The Golgi resident protein GM130 remained in the perinuclear region regardless of CFP-TMEM30A transfection (Fig 7D). Although TGN38 was mainly distributed in the perinuclear region of APP-Venus transfected cells, its distribution dramatically shifted to the enlarged vesicular compartments that contained APP-Venus in CFP-TMEM30A-cotransfected cells (Fig 7E). Although these traffic impairments are not limited to endosomes and may affect overall vesicular traffics, our data indicated that the formation of a complex between TMEM30A and FL-APP/βCTF is initiated in endosomes and mainly results in their defective maturation and trafficking.

### TMEM30A facilitates Aβ production following high BACE1 expression levels

In light of APP metabolism, our results suggested that TMEM30A reduced β-cleavage but induced APP-CTF accumulation through endosomal traffic impairment. We considered that TMEM30A-induced endosomal abnormalities caused the mislocalization of endogenous BACE1 and impaired β-cleavage. Because COS-7 cells express very low endogenous BACE1 levels compared with neuronal cells, we assessed the trafficking and activity of newly synthesized BACE1 in APP-TMEM30A-expressing cells.

**Fig 8:**
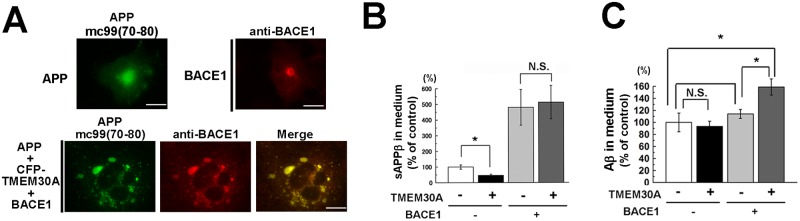
Effect of BACE1 expression in APP-TMEM30A transfected cells. A–C: COS-7 cells were transfected with CFP-TMEM30A and APP. After 24 h of transfection, cells were further transfected with BACE1. After 24 h of BACE1 transfection, the medium was refreshed, and cells were further incubated for 24 h. (A) Immunofluorescence analysis. Cells were labeled with anti-APP C-terminal antibody [mC99 (70–80)] and anti-BACE1 C-terminal antibody. Scale bar: 20 μm. (B) Quantification analysis of sAPPβ levels in the medium by immunoblotting (independent experiments were performed thrice (n = 3), mean ± SEM; *P < 0.05; N.S., no significant difference). (C) Quantification of secreted Aβ by ELISA (n = 3, mean ± SEM).

In control cells, BACE1 was distributed in perinuclear and small vesicular structures. In contrast, in APP-TMEM30A cotransfected cells, newly synthesized BACE1 accumulated in enlarged vesicles containing APP (Fig 8A). Although the coexpression of TMEM30A and APP reduced sAPPβ (Fig 5), further BACE1 expression restored sAPPβ levels in TMEM30A-expressing cells (Fig 8B). We observed that additional BACE1 expression caused an increase in Aβ production compared with BACE1-transfected cells without TMEM30A expression (Fig 8C).

## Discussion

Here we identified TMEM30A as a candidate partner for FL-APP/βCTF within endosomes. The interaction between TMEM30A and FL-APP/βCTF may have induced abnormally enlarged endosomes, which accumulated the early endosome marker Rab5 (Fig 1A–1C), late endosome marker Rab7, and recycling endosome marker Rab11 (Fig 7A and 7B). These results are similar to the accumulation of various Rab markers in enlarged endosomes that have been observed in the brains of aged monkeys [6], as well as in the brains of patients with AD and DS [5, 35]. Furthermore, we observed that TGN38, a TGN resident protein that cycles between the Golgi and plasma membrane through endosomes by retrograde traffic, was mislocalized in enlarged endosomes that were induced by APP-TMEM30A coexpression (Fig 7E). Our data suggested that the complex formation between TMEM30A and FL-APP/βCTF in endosomes induced impairment in retrograde and recycling traffic, as well as disturbance in endosomal maturation, consistent with observations in the early AD stages.

Based on the results obtained using APP deletion mutants (Fig 3) and the GST pulldown assay (Fig 5), APP was hypothesized to interact with TMEM30A through the following two domains: the N-terminal region from β-cleavage site of APP and Aβ N-terminal sequence. This hypothesis is supported by the finding that TMEM30A interacts with FL-APP and βCTFs but not with αCTF that lacks an Aβ N-terminal sequence (Figs 2B and 4C). In enlarged endosomes, TMEM30A was highly co-localized with βCTF specific staining, but less with FL-APP (Fig 2C), consistent with the evidence that βCTF is more abundant in endosomes [29]. In contrast, the ER resident KKLN mutant of TMEM30A failed to both interact with βCTFs (Fig 4C) and induce enlarged endosomes (Fig 4D). These data indicated that the interaction between TMEM30A and βCTFs in endosomes may be key for vesicular traffic impairment and enlarged endosomal morphology.

The effect of TMEM30A on APP metabolism is complex. Although the cotransfection of TMEM30A increased FL-APP and APP-CTF levels, sAPPβ levels decreased (Fig 6). Moreover, the cotransfection of APP and TMEM30A led to βCTF accumulation but failed to alter Aβ production. Considering that APP-CTFs are metabolized by cleavage of γ-secretase or by lysosomal degradation pathways [36] and that endosomal traffic impairment often reduces lysosomal degradation of proteins [6], we hypothesized that the TMEM30A-βCTF complex formation impaired endosomal traffic to block the transport of APP metabolites to lysosomes and γ-secretase. Further studies should analyze the involvement of endosomal traffic and the effects of TMEM30A on γ-secretase and lysosomal activities.

A part of our data is consistent with previous findings that the impairment of retrograde traffic results in decreased β-secretase cleavage of APP in cultured cells [37]. These cell lines show reduced BACE1 expression compared with that in neurons. Thus, BACE1 cleavage and Aβ production might be mainly dependent on APP trafficking. However, BACE1 traffic is an important regulatory factor for APP metabolism in neuronal cells that strongly express BACE1. This idea could be supported by the finding that the expressed BACE1 accumulated in enlarged vesicles formed by APP and TMEM30A (Fig 8A). This restored sAPPβ levels, which were reduced by TMEM30A expressions (Fig 8B). Most importantly, Aβ production increased under this condition (Fig 8C). Our data suggested that the regulatory mechanisms of TMEM30A primarily depended on the spatial regulation of APP and BACE1 and that newly synthesized BACE1 colocalized with APP, thereby enhancing the accumulation of APP-CTFs and increasing Aβ production.

How does the interaction between TMEM30A and APP/βCTF in endosomes affect vesicular traffic? We believe that two possibilities exist. First, the TMEM30A-APP/βCTF complex formation impairs the endogenous functions of lipid flippase to regulate PS distribution. The asymmetric distribution of PS in lipid bilayers regulates the budding of the vesicular cargo [18] and recruits PS-interacting vesicular traffic regulators. The dysfunction of the TMEM30A and P4-ATPase homologs in *C*. *elegans* resulted in the accumulation of endosomes and mislocalization of PS [19, 20]. In addition, the asymmetrical distribution of PS is important for the retrograde traffic from the plasma membrane to TGN via recycling endosomes [16, 17, 28]. Because ATP8A1 was excluded from the complex comprising TMEM30A and APP (Fig 2B), our data suggested that βCTF and P4-ATPase compete for TMEM30A to reduce lipid flippase activity. Further analyses of the effect of βCTF on intracellular PS distributions and activity and/or localization of P4-ATPases are required.

The other possibility is that TMEM30A stabilizes βCTF in endosomes to trap regulators that are essential for endosomal transport. The abnormal activation of Rab5 itself causes endosomal accumulation [35], and Rab5 accumulates in abnormal early endosomes in AD neurons [38]. Rab5 accumulation in enlarged endosomes (Fig 1C) may reflect the complex comprising βCTF and signaling molecules in endosomes [4]. Supporting our hypothesis, βCTFs is reported to function as a Rab5 activator [39]. Additionally, βCTFs accumulated in endosomes to form complexes with Rab5 and a Rab5 effector APPL1 [40]. In addition to APPL1, we are currently searching for endosomal proteins that interact with the complex of TMEM30A and βCTFs.

In this study, we identified TMEM30A as a novel modulator for APP trafficking and metabolism. Moreover, we showed that TMEM30A is a candidate partner for βCTF in endosomes and may cause endosomal dysfunction. Considering that the newly synthesized BACE1 accumulated in enlarged endosomes to overproduce Aβ, we proposed a novel role for TMEM30A in endosomal traffic impairment that is responsible for Aβ overproduction. Compounds that promote retrograde trafficking reduce Aβ production [41], suggesting that AD-specific traffic impairment is a promising therapeutic approach for AD. Our study suggested that inhibiting the interaction between TMEM30A and βCTF and the resultant endosomal traffic impairment is a possible target for alleviating Aβ production.

## Supporting information

S1 File Supplemental methods. (DOCX)

S1 Fig Quantitative analysis of codistribution between APP-Venus and Rab5. A: Quantitative analysis of APP-Venus and Rab5. Staining conditions were similar to those in Fig 1C. At least 10 cells from each sample were analyzed, and each independent experiment was performed in triplicate (*n* = 3 mean ± SEM, ***p < 0.001 by Student's t test.). B: Staining of Rab5 was performed using anti-Rab5 (Synaptic Systems). Note that the staining pattern is comparable to that in Fig 1C. Scale bar: 10 μm.
(TIF)

S2 Fig Coaccumulation of TMEM30A and APP in transfected primary hippocampal neurons. Rat primary hippocampal neurons were transfected with APP-EGFP and/or mCherry-TMEM30A. Transfection was carried out 9 d after plating and cells were fixed 2 d after transfection. Scale bar: 20 μm.
(TIF)

S3 Fig Schematic view of APP metabolites and antibodies used in this study. Schematic depiction of the APP metabolites produced by cleavage of α- β-, and γ-secretases. Antibodies used for detection of each APP metabolite are shown.
(TIF)

S4 Fig Interaction between TMEM30A and APP-CTFs in neuroblastoma cell lines. A: BE(2)-C cells were transfected with CFP-TMEM30A. After 24 h of transfection, cells were treated with BACE1 inhibitor IV (10 μM) for 24 h. Note that TMEM30A expression accumulated APP-CTFs derived from endogenous APP. B: Normal SH-SY5Y cells (-) and cells stably expressing Swedish mutant of APP (Swe) were subjected to immunoprecipitation using control IgG (C) or TMEM30A antibody (T). APP-CTFs were co-immunoprecipitated with endogenous TMEM30A. C: Iodixanol fractionation was performed using SH-SY5Y cells. Distribution of the following markers across the gradient was analyzed: Rab5 (endosome), GM130 (TGN), and Calreticulin (CRT, ER).
(TIF)

S5 Fig Cotransfection of TMEM30A and APP-Venus accumulated mCherry tagged Rab proteins in enlarged endosomes. COS-7 cells were cotransfected with APP-Venus and TMEM30A. After 24 h, cells were transfected with mCherry-Rab5, Rab7 or Rab11. After 24 h, cells were fixed and immunofluorescence images were captured with Keyence fluorescence microscope BZ-X700 by using Haze Reduction function.
(TIF)
