# Supplementary material for: TMEM30A is a candidate interacting partner for the β-carboxyl-terminal fragment of amyloid-β precursor protein in endosomes
Source: PLoS One. 2018 Aug 7;13(8):e0200988. doi: 10.1371/journal.pone.0200988 (PMC6080755; doi:10.1371/journal.pone.0200988)
Supplement: S1 File — (DOCX) [file pone.0200988.s001.docx]

**S1 Supplemental methods**

**Additional materials**

Following reagents were purchased: β-secretase inhibitor IV (Merck Millipore), anti-Rab5 (108011, Synaptic Systems), and anti-Calreticulin (Stressgen).

**Codistribution analysis**

Codistribution analysis of immunofluorescence images was performed using JacoP plugin of Image J [1]. Staining conditions were identical to those mentioned in Fig 1C. At least 10 cells were analyzed within each sample and each independent experiment was performed in triplicate.

**Preparation and transfection of rat primary hippocampal neurons**

Primary hippocampal neurons were prepared from hippocampi of embryonic day 20 Sprague–Dawley rats using a previously described method [2]. Cells isolated by trypsinization and trituration were plated onto 0.1% polyethyleneimine-coated glass coverslips in 12-well plates (15-mm diameter, 2*10^5^ cells/coverslip). Cells were maintained in Neurobasal medium supplemented with 2.5 mM GlutaMax and 2% B-27 (Thermo Fisher). Transfection was carried out 9 d after plating using a TransMessenger Transfection Kit (Qiagen) with 1.2 μg of expression vectors, pcDNA3-APP-EGFP, and/or pcDNA-mCherry-TMEM30a. After 2 d, the cells were fixed with 4% paraformaldehyde in phosphate-buffered saline (PBS) and mounted. The fluorescent images were captured using an inverted microscope (IX71 with a water-immersion 60X objective lens, Olympus, Japan) equipped with a multi-band filter set (DA/FI/TR/Cy5, Semrock) and Orca-ER cooled CCD camera (Hamamatsu Photonics, Japan). This experiment was approved by the committees on animal experiments of Juntendo University and performed according to the institutional guidelines (please refer to Materials and Methods section in the manuscript).

**Additional cultured cell lines used in this study and establishment of stable cell lines**

Human neuroblastoma cell lines, SH-SY5Y (ATCC CRL-2266) and BE(2)-C (ATCC CRL-2268) were used. Swedish mutant of APP was introduced to episomal plasmid (pEB-hyg, Wako). After transfecting this plasmid in SH-SY5Y, cells were selected on Hygromycin (Wako).

**Iodixanol gradient fractionation**

Cells were washed with cold PBS and subsequently homogenized in homogenization buffer (HB, 250 mM Sucrose, 20 mM Tris-HCl (pH 7.4), 1 mM EGTA, 1 mM EDTA, and protease inhibitors) by Dounce homogenizer and by passing through a 21-G needle 20 times. Lysate was incubated on ice for 10 min and postnuclear supernatant was collected after centrifugation at 825 x g for 15 min at 4°C. Supernatant was mixed with an identical volume of 50% Optiprep (Alere Technologies AS) in HB. Two ml of the mixture was placed at the bottom of an ultracentrifuge tube (14*89 mm) and overlaid gently with 1 ml each of 20%, 18.5%, 16.5%, 14.5%, 12.5%, 10.5%, 8.5%, 6.5%, and 5% Optiprep in HB. After centrifugation at 125,000 x g (27000 rpm) for 20 h at 4°C (SW41, Beckman Coulter), 500 µL fractions were collected from the top and trichloroacetic acid (TCA, Wako) precipitation was performed. Final TCA concentration was adjusted to 15% and the mixture was incubated on ice for 30 min. After subsequent centrifugation at 20,000 x g for 15 min at 4°C, the precipitate was dissolved in RIPA buffer, mixed with SDS-sample buffer, and analyzed by immunoblotting.

1. Bolte S, Cordelières FP. A guided tour into subcellular colocalization analysis in light microscopy. Journal of Microscopy. 2006;224(3):213-32. doi: 10.1111/j.1365-2818.2006.01706.x.

2. Sakurai T, Kaneko K, Okuno M, Wada K, Kashiyama T, Shimizu H, et al. Membrane microdomain switching: a regulatory mechanism of amyloid precursor protein processing. The Journal of cell biology. 2008;183(2):339-52. doi: 10.1083/jcb.200804075. PubMed PMID: 18936252; PubMed Central PMCID: PMC2568028.
