# Supplementary figures and images for: TMEM30A is a candidate interacting partner for the β-carboxyl-terminal fragment of amyloid-β precursor protein in endosomes
Source: PLoS One. 2018 Aug 7;13(8):e0200988. doi: 10.1371/journal.pone.0200988 (PMC6080755; doi:10.1371/journal.pone.0200988)

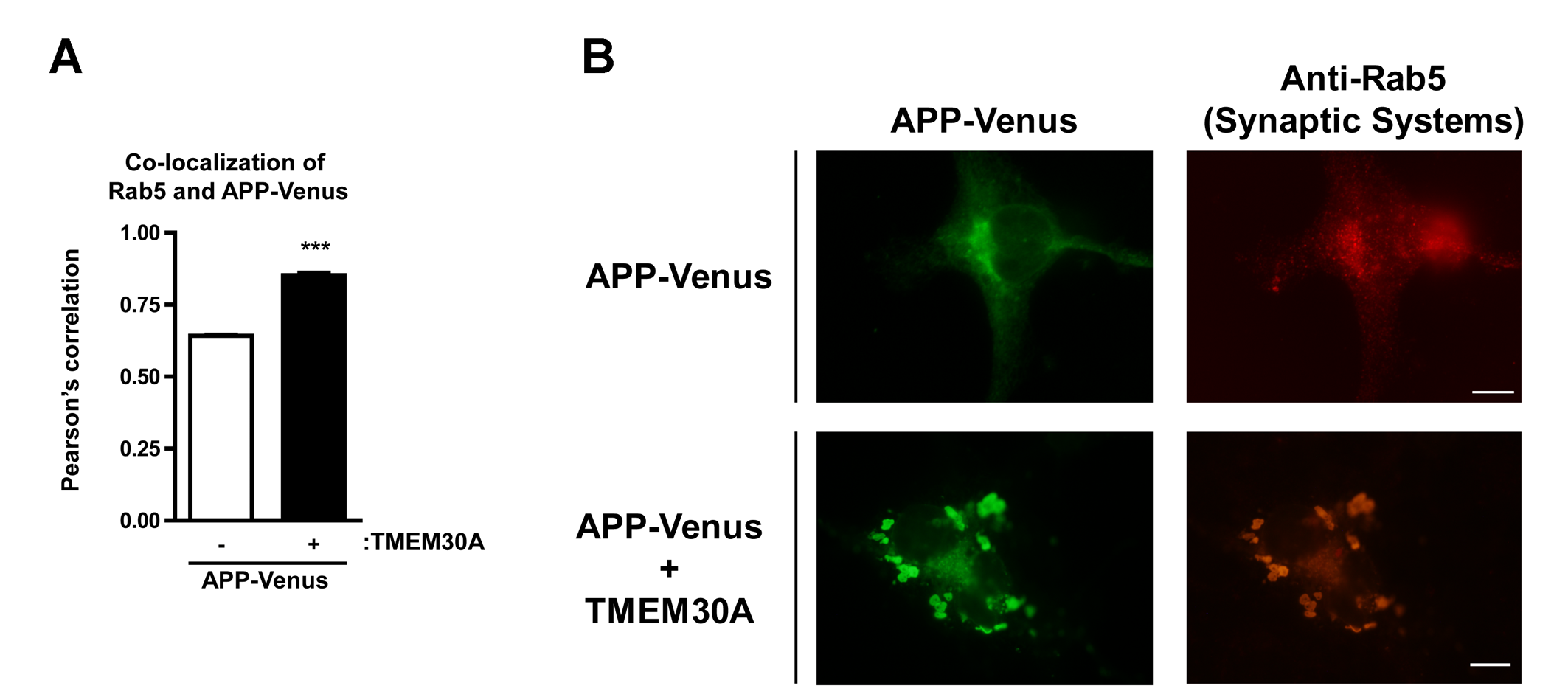

Supplement: S1 Fig — A: Quantitative analysis of APP-Venus and Rab5. Staining conditions were similar to those in Fig 1C. At least 10 cells from each sample were analyzed, and each independent experiment was performed in triplicate (n = 3 mean ± SEM, ***p < 0.001 by Student's t test.). B: Staining of Rab5 was performed using anti-Rab5 (Synaptic Systems). Note that the staining pattern is comparable to that in Fig 1C. Scale bar: 10 μm. (TIF) [file pone.0200988.s002.tif]

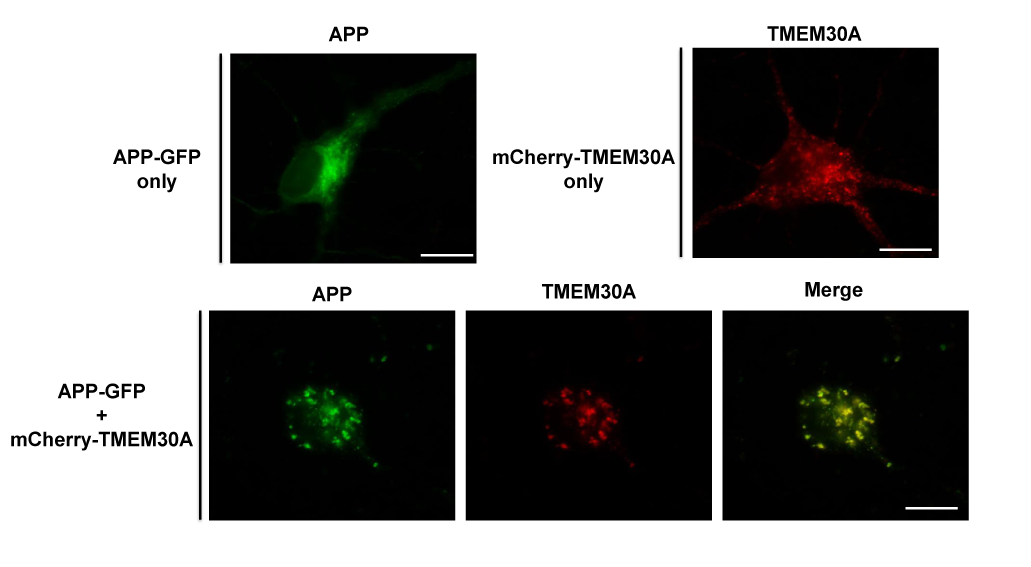

Supplement: S2 Fig — Rat primary hippocampal neurons were transfected with APP-EGFP and/or mCherry-TMEM30A. Transfection was carried out 9 d after plating and cells were fixed 2 d after transfection. Scale bar: 20 μm. (TIF) [file pone.0200988.s003.tif]

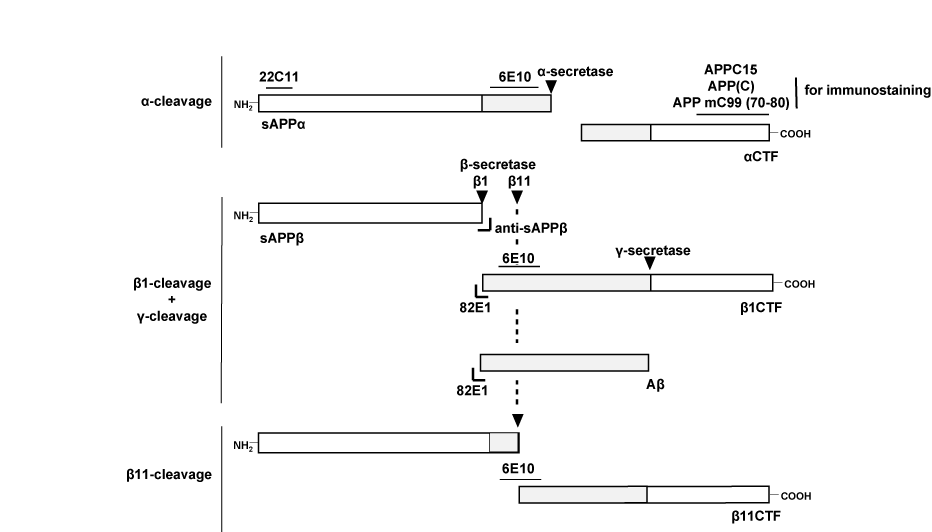

Supplement: S3 Fig — Schematic depiction of the APP metabolites produced by cleavage of α- β-, and γ-secretases. Antibodies used for detection of each APP metabolite are shown. (TIF) [file pone.0200988.s004.tif]

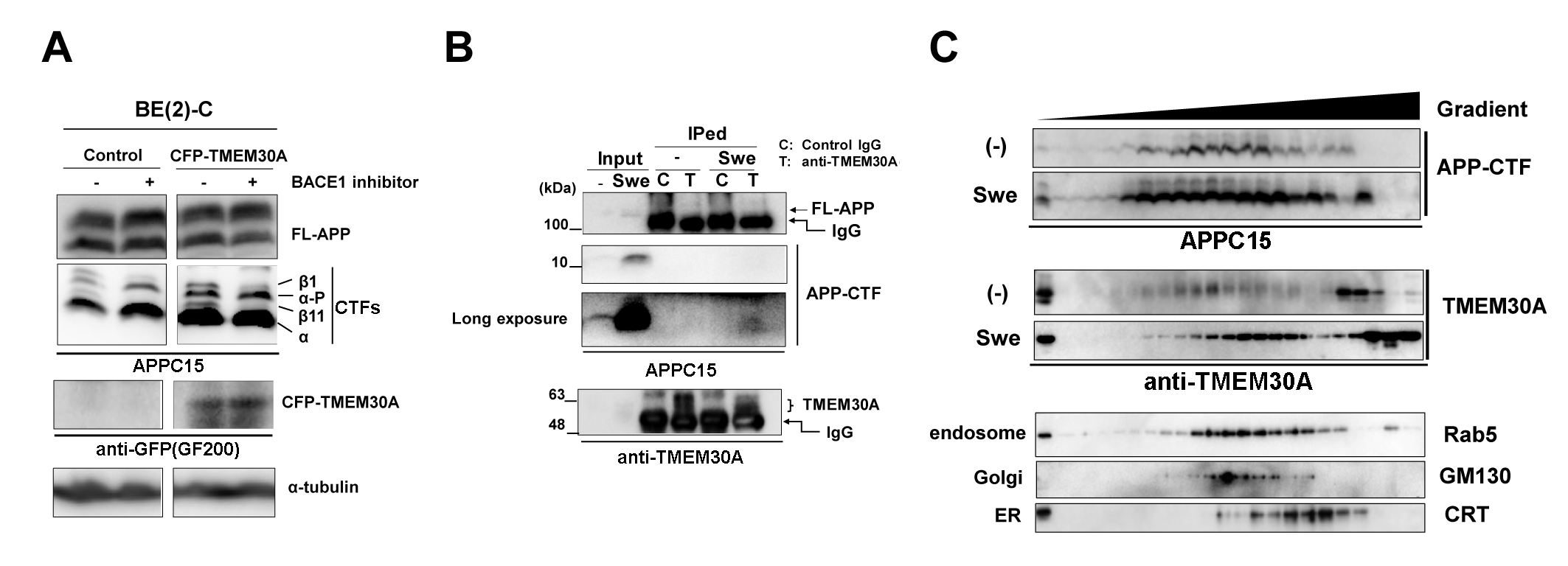

Supplement: S4 Fig — A: BE(2)-C cells were transfected with CFP-TMEM30A. After 24 h of transfection, cells were treated with BACE1 inhibitor IV (10 μM) for 24 h. Note that TMEM30A expression accumulated APP-CTFs derived from endogenous APP. B: Normal SH-SY5Y cells (-) and cells stably expressing Swedish mutant of APP (Swe) were subjected to immunoprecipitation using control IgG (C) or TMEM30A antibody (T). APP-CTFs were co-immunoprecipitated with endogenous TMEM30A. C: Iodixanol fractionation was performed using SH-SY5Y cells. Distribution of the following markers across the gradient was analyzed: Rab5 (endosome), GM130 (TGN), and Calreticulin (CRT, ER). (TIF) [file pone.0200988.s005.tif]

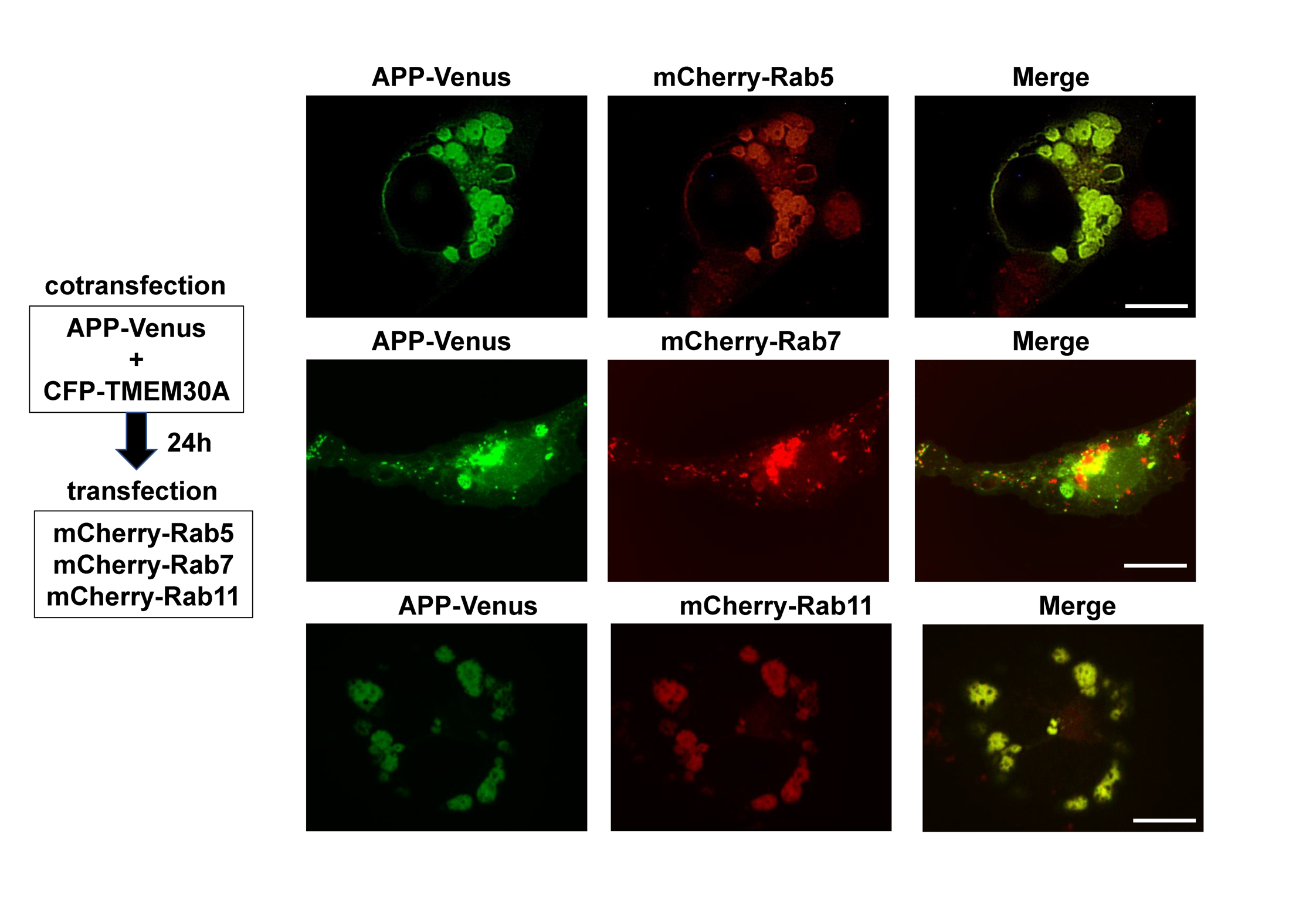

Supplement: S5 Fig — COS-7 cells were cotransfected with APP-Venus and TMEM30A. After 24 h, cells were transfected with mCherry-Rab5, Rab7 or Rab11. After 24 h, cells were fixed and immunofluorescence images were captured with Keyence fluorescence microscope BZ-X700 by using Haze Reduction function. (TIF) [file pone.0200988.s006.tif]
